# The practice of using repurposed medications as chemoprophylaxis for COVID-19 by healthcare workers in a tertiary hospital in Southern Nigeria

**DOI:** 10.4314/ahs.v23i2.5

**Published:** 2023-06

**Authors:** Sylvanus B Udoette, Asukwo E Onukak, Victor A Umoh, Akpabio A Akpabio

**Affiliations:** Department of Internal medicine, University of Uyo teaching hospital, Uyo, Akwa Ibom state, Nigeria

**Keywords:** COVID-19, chemoprophylaxis, healthcare workers

## Abstract

**Background:**

Coronavirus disease 2019 (COVID-19) is a viral infection that has been reported in virtually every country. Healthcare workers (HCW) are more at risk of COVID-19 than the general population making them a priority group for vaccination. Before the roll out of COVID-19 vaccines in Nigeria, some HCW were using some repurposed, unapproved drugs to possibly prevent the disease. This study evaluated the frequency and pattern of drug use for COVID-19 prevention.

**Methods:**

This was a prospective cross-sectional study of HCW conducted in Southern Nigeria. Data was obtained from the participants using a questionnaire and blood sample was obtained for SARS-CoV-2 antibody testing. Data was analysed using the statistical package for social sciences (SPSS) version 23.

**Results:**

One hundred and sixty-six participants were enrolled in this study. Thirty-two (19.3%) of them had taken a repurposed medication as prophylaxis for COVID-19. The most used drugs were Vitamin C (9%), Azithromycin (8.4%) and Zinc (6.6%). History of contact with patient with confirmed COVID-19 and being a pharmacist were independent factors associated with the use of COVID-19 prophylaxis.

**Conclusion:**

Several HCW in Nigeria take drugs to possibly prevent COVID-19. These medications may not offer significant protection against COVID-19. There is an urgent need to increase uptake of COVID-19 vaccines in HCW in Nigeria.

## Introduction

Coronavirus disease 2019 (COVID 19) is a multi-systemic viral infection that can be fatal. The disease is caused by Severe Acute Respiratory Syndrome Coronavirus- 2 (SARS-CoV-2). It was first reported in Wuhan, China in December 2019 and since then the infection has spread to almost all countries affecting over 40 million people and causing over 1 million deaths as at November 1,2020. 1-2 These numbers may underestimate the actual burden of the disease as there is insufficient testing capacity in many countries.^3^ Apart from these health consequences, COVID- 19 has also caused social and economic disruptions globally.^4^

Healthcare workers (HCW) have a significantly higher risk of COVID 19 than the general population.^3^ The infection rate in healthcare workers varies in different countries with rate of 4.4% in China and 20% in Italy.^5^ Healthcare workers have a higher risk of infection with COVID-19 because apart from the possibility of acquiring infection due to their interactions with patients in the hospital, they also have a risk of being infected while in the community.^6^ While they are at work, HCW spend a long time with patients who may have COVID-19, they may have inadequate personal protective equipment and sometimes use a personal protective equipment that has poor quality.^6^ Measures to mitigate this risk in HCW is urgently needed especially in Africa where the numbers of HCW are limited. Isolation of HCW who become infected with the virus or following high risk exposure imposes a further burden on limited manpower available in the continent.^4^ Since HCW may be the source of infection to their family members and patients, reduction of the rate of infection in HCW will help reduce the spread of COVID-19.6 The world health organisation recommends the use of non-pharmacological approach for the prevention of infections in healthcare settings.^6^

In view of the impact of COVID 19 on the lives and livelihood of people worldwide, a wide range of non-pharmacological and pharmacological prevention strategies have been used in controlling this pandemic.^7^ Most of the prevention strategies have utilized non-pharmacological approaches such as the use of face mask, hand hygiene, social distancing, quarantine and isolation. Implementation of these strategies has some limitations.^3^ Availability of a safe and effective vaccine is likely to be the most appropriate pharmacological approach for the prevention of COVID 19.^7^ However, development and distribution of vaccines took months and use of available drugs as a stop gap measure was an attractive option.^3^,^7^

Some medications such as chloroquine, hydroxychloroquine, zinc, ivermectin, vitamin C and azithromycin that have already been in use were repurposed and tried off-label as prophylaxis for COVID 19 even when there is no strong evidence that they work.^1^,^7^-^9^ Vitamin C is an essential micronutrient that serves as a scavenger of free radicals. It also supports cellular functions thereby enhancing both innate and adaptive immune response thereby reducing susceptibility to some viral infections.8 Ivermectin is an inhibitor of importin-α/β-mediated nuclear import. This action leads to the reduction of nuclear transport of viral proteins and suppression of the replication of some RNA viruses such as yellow fever and SARS-CoV-2.^9^ Regulatory authorities in most countries (apart from India) never recommended the use of hydroxychloroquine or other repurposed medications as prophylaxis for COVID 19 in HCW and close contacts of confirmed cases of COVID 19.^4^

Although the use of repurposed medications as chemoprophylaxis may look attractive, there are some drawbacks. Some of these medications being used to possibly prevent COVID-19 has adverse cardiovascular effects.^10^ Healthcare workers are wasting meagre income on medications with no proven efficacy.^11^-^12^ Use of these medications may give false sense of security without necessarily reducing risk of acquiring and transmitting COVID-19.^4^ Off-label use of antimicrobials can worsen the antimicrobial resistance crisis leading to poor health outcomes.^13^

Vaccination of HCW for COVID-19 started in March 2021. This study, conducted at the time when there were no COVID-19 vaccines in Nigeria evaluated the prevalence of the use of medications as prophylaxis for COVID 19 among HCW as well as the type of medications used for COVID 19 prophylaxis.

## Methodology

### Study design

This was a descriptive cross-sectional study.

### Study setting/duration

This study was conducted at a tertiary hospital located in Southern Nigeria over a 1-month period from July 1 to July 31, 2020.

### Study population/sampling method

All the participants in this study were healthcare workers (doctors, nurses, pharmacists, physiotherapists, cleaners, administrative staff, medical laboratory scientists and radiographers) randomly selected from the hospital's nominal records. This was done by randomizing the staff master list in Microsoft Excel version 2016 then selecting the first 166. Selected persons were called over the phone and consecutively enrolled in the study. Whenever a selected person did not consent to participate, the next person on the list was invited to participate until the proposed sample size of 166 was attained.

### Ethical considerations

Ethical approval for this study was obtained from the Ethical committee of the hospital before the commencement of this study. Written, informed consent was obtained from every participant enrolled in this study.

### Data collection/Handling

A questionnaire was used to obtain sociodemographic details of participants, history of use of drugs for COVID-19 chemoprophylaxis and history of contact with patient with COVID-19. Blood sample was collected (using finger prick) from all the participants for SARS-CoV-2 serology and the results were recorded on the questionnaire. This test detects antibodies (IgG or IgM) to SARS-CoV-2 in patients' serum using enzyme-linked immunosorbent assay method.

### Definition of terms

Seropositivity was defined as the presence of either IgM or IgG (or both) antibodies in the serum of the participants. Chemoprophylaxis was defined as the use of any medication with the aim of preventing COVID-19 whether as a pre-exposure prophylaxis or a post-exposure prophylaxis.

Data analysis was done using the statistical package for social sciences IBM version 23. The continuous variables were described using mean and standard deviation whereas the categorical variables were presented as percentages. The categorical variables were compared using chi-square or Fisher's exact test when indicated. Multivariate logistic regression was done to identify factors independently associated with use of prophylaxis for COVID-19

## Results

A total of 166 participants comprising 59 males and 107 females were enrolled in this study. All the participants were healthcare workers. Thirty-two (19.3%) of them had taken prophylaxis for COVID-19. Twelve out of the 59 male (20.3%) participants took prophylaxis while 20 out of the 107 female (18.7%) participants took prophylaxis. This difference was not statistically significant (p = 0.838). The participants who took medications as a prophylaxis for COVID-19 and those who did not did not differ by age, marital status and staff cadre. There was also no statistical difference between the SARS-CoV-2 IgG serostatus of participants who took medications for chemoprophylaxis and those who did not (p = 0.397). Participants who have had contact with patients with COVID-19 were more likely to take prophylaxis compared to those who had no history of contact with COVID-19 patients (p< 0.005). The characteristics of participants in this study are shown in Table 1.

**Table 1:** Characteristics of the study population

	Total (N = 166) n (%)	Prophylaxis users (N = 32) n (%)	Nonusers of prophylaxis (N = 134) n (%)		
Variable				X^2^/t	P value
**Age (years)**					
< 30	15 (9)	5 (15.6)	10 (7.5)	3.014	0.389
30 - 39	89 (53.6)	17 (53.1)	72 (53.7)		
40 - 49	42 (25.3)	8 (25)	34 (25.4)		
50-59	20 (12)	2 (6.2)	18 (13.4)		
**Gender**					
Female	107 (64.5)	20 (62.5)	87 (64.9)	0.066	0.797
Male	59 (35.5)	12 (37.5)	47 (35.1)		
*Marital status					
Married	122 (73.5)	23 (71.9)	99 (73.9)		0.736
Single	41 (24.7)	8 (25)	33 (24.6)		
Separated	3 (1.8)	1 (3.1)	2 (1.5)		
*Staff Cadre					
Doctors	54 (32.5)	16 (50)	38 (28.4)		0.053
Nurses	53 (31.9)	9 (28.1)	44 (32.8)		
Laboratory staff					
Pharmacists	18 (10.8)	2 (6.3)	16 (11.9)		
Others	4 (2.4)	2 (6.3)	2 (1.5)		
Cleaner	33 (19.9)	2 (6.3)	31 (23.1)		
	4 (2.4)	1 (3.1)	3 (2.2)		
**Contact with COVID-19 patient**					
Yes	79 (47.6)	27 (84.4)	52 (38.8)	21.506	**<0.005**
No	87 (52.4)	5 (15.6)	82 (61.2)		

The number in bold represent significant value, X2/t = chi square, * = Fisher exact test, Others include physiotherapists, administrative staff, radiographers and health information managers

The most used drug for COVID-19 prophylaxis were vitamin c (9%), azithromycin (8.4%), zinc (6.6%), chloroquine (6%) and hydroxychloroquine (3%). The various drugs used by the participants for COVID-19 prophylaxis is shown in Table 2. These drugs were used in varying combinations. Twelve (7.2%) of the participants used only one drug, 11 (6.6%) used 2 drugs while 6 (3.6%) used 3 drugs.

**Table 2:** Drugs used by participants for COVID-19 prophylaxis

Drug	Number of participants who used the drug
	n (%)
Amoxicillin/Clavulanic acid	1 (0.6)
Vitamin E	2 (1.2)
Herbal preparations	3 (1.8)
Hydroxychloroquine	5 (3.0)
Chloroquine	10 (6.0)
Zinc	11 (6.6)
Azithromycin	14 (8.4)
Vitamin C	15 (9.0)

Following multivariate logistic regression, being a pharmacist and contact with someone with COVID-19 were independent risk factors associated with use of prophylaxis by healthcare workers. The multivariate logistic model is shown in Table 3.

**Table 3:** Multivariate logistic regression model for factors associated with use of COVID-19 prophylaxis

Variable	B	P value	Exp (B)	95% C.I for Exp (B)
Gendre	-0.333	0.570	0.717	0.262-1.960
Cadre				
Nurses	-0.829	0.161	0.436	0.137-1.392
Lab. Scientists	-0.709	0.414	0.492	0.090-2.702
**Pharmacists**	**2.483**	**0.038**	**11.974**	**1.143-125.491**
Cleaners	-0.336	0.785	0.715	0.064-8.001
Others	-19.400	0.998	0.000	
Admin staff	-18.591	0.999	0.000	
Physiotherapists	-0.850	0.391	0.427	0.061-2.975
Daily Exposure	-0.191	0.251	0.826	0.594-1.149
**Contact with COVID-19 patient**	**2.354**	**0.001**	**10.532**	**2.759-40.205**
Age	0.002	0.960	1.002	0.938-1.070

Variables with significant values are written in bold

## Discussion

Our study showed that some healthcare workers in Nigeria were taking some medications with the aim of preventing COVID-19 even when these medications have not been approved for the purposes of either pre-exposure or post-exposure prophylaxis for COVID-19.^10^-^11^ This practice was observed among healthcare workers of various cadres. The use of these drugs may offer false sense of protection resulting in increase in infection rates in hospitals.^4^ The use of some drugs for COVID-19 prophylaxis is probably driven by fear and media influence and not necessarily medical evidence.^7^,^14^-^16^

The proportion of healthcare workers who had taken prophylaxis for COVID-19 in this study (19.3%) was lower than what was reported by Dhamija et al (35.5%). The later study involved a worldwide survey of healthcare with most of the respondents working in India.^17^ The health authorities in India initially recommended the use of chloroquine or hydroxychloroquine for prophylaxis against COVID-19.^17^ A study conducted among HCW in Southwest Nigeria reported that 19% of them had taken antibiotics as self medication. This figure is similar to the finding in our study.^18^

In our study, pharmacists were 11 times more likely to use chemoprophylaxis for COVID-19 compared to doctors. This may have been due to the ease of assessing these medications. Another study had reported that pharmacist trainees were more likely to self-prescribe antibiotics than non-pharmacy students.^19^ The HCW in our study who had contact with patients with COVID-19 were 10 times more likely to use drugs for COVID-19 prophylaxis compared to other workers. Healthcare workers who have had contact with patients with COVID-19 are more likely to be infected than others and may explain why they would want to use drugs as post-exposure prophylaxis for COVID-19.^6^ The widespread use of these medications especially antibiotics like azithromycin for COVID-19 prophylaxis can potentially increase the burden of antimicrobial resistance in Nigeria. This will in turn lead to increase health expenditure and poor patients' outcomes.^13^ Some of the drugs used by the participants in this study have adverse cardiovascular side effects.^10^ The cardiotoxicity caused by chloroquine and hydroxychloroquine may be augmented by concomitant administration of cytochrome P-450 enzyme inhibitors like azithromycin.^4^

Healthcare workers are very important in efforts to combat COVID-19, they are also more at risk of acquiring the infection. Besides the high risk of infection with SARS-CoV-2, HCW are confronted with other challenges such as exhaustion, difficult triage decisions, pains of losing colleagues and patients.^3^ They should not be allowed to depend solely on drugs that may not reduce their risk of having COVID-19. Healthcare workers are therefore a priority group that should be offered COVID-19 vaccine. The use of prophylaxis did not change the SARS-CoV-2 serostatus of the participants of this study. The major challenge with the use of chloroquine and hydroxychloroquine has been the non-translation of in vitro successes to in vivo impact.^12^, ^14^ This is similar to what was observed with chloroquine and influenza. Prophylaxis in influenza failed even when chloroquine had in vitro efficacy against the virus.4 This might probably imply that they may not be effective for this purpose. There is a need for further randomised controlled trials to determine which readily available drug can be repurposed to serve as chemoprophylaxis for COVID-19.

Since there is no convincing in vivo and clinical evidence yet, it would be unadvisable to recommend these drugs for prophylaxis in COVID-19. Vaccines and non-pharmacological options such as personal hygiene, use of face mask and physical distancing remain the best preventive strategy.^11^

## Limitations

The relatively small sample size of this study and the fact that it was a single-centre study are the limitations of this study. The spectrum of drugs used by HCW for COVID-19 prophylaxis may be different in other health facilities in Nigeria making it difficult to extrapolate some of the findings of this study.

## Conclusion

Healthcare workers in Nigeria were taking several medications hoping to prevent COVID-19 especially when vaccines were not available in the country putting them at risk of drug adverse effects without necessarily reducing the probability of being infected with COVID-19 as these agents may not very effective.
